# Effectiveness of Internet-Based Cognitive Behavioral Therapy With Telephone Support for Noncardiac Chest Pain: Randomized Controlled Trial

**DOI:** 10.2196/33631

**Published:** 2022-01-24

**Authors:** Terje Thesen, Joseph A Himle, Egil W Martinsen, Liv T Walseth, Frode Thorup, Frode Gallefoss, Egil Jonsbu

**Affiliations:** 1 Distriktspsykiatrisk senter Solvang Sørlandet Hospital Sørlandet sykehus Helse Foretak Kristiansand Norway; 2 Faculty of Medicine and Health Science Norwegian University of Science and Technology Trondheim Norway; 3 School of Social Work and School of Medicine-Psychiatry University of Michigan Ann Arbor, MI United States; 4 Institute of Clinical Medicine University of Oslo Oslo Norway; 5 Department of Cardiology Sørlandet Hospital Sørlandet sykehus Helse Foretak Kristiansand Norway; 6 Faculty of Medicine University of Bergen Bergen Norway; 7 Department of Clinical Research Sørlandet Hospital Sørlandet sykehus Helse Foretak Kristiansand Norway; 8 Department of Psychiatry Møre and Romsdal Hospital Trust Molde Norway

**Keywords:** noncardiac chest pain, internet-based treatment, internet-assisted treatment, cognitive behavioral therapy, psychosomatic medicine, randomized controlled trial, pain, treatment, internet-based cognitive behavioral therapy, effectiveness, support, intervention

## Abstract

**Background:**

Noncardiac chest pain has a high prevalence and is associated with reduced quality of life, anxiety, avoidance of physical activity, and high societal costs. There is a lack of an effective, low-cost, easy to distribute intervention to assist patients with noncardiac chest pain.

**Objective:**

In this study, we aimed to investigate the effectiveness of internet-based cognitive behavioral therapy with telephone support for noncardiac chest pain.

**Methods:**

We conducted a randomized controlled trial, with a 12-month follow-up period, to compare internet-based cognitive behavioral therapy to a control condition (treatment as usual). A total of 162 participants aged 18 to 70 years with a diagnosis of noncardiac chest pain were randomized to either internet-based cognitive behavioral therapy (n=81) or treatment as usual (n=81). The participants in the experimental condition received 6 weekly sessions of internet-based cognitive behavioral therapy. The sessions covered different topics related to coping with noncardiac chest pain (education about the heart, physical activity, interpretations/attention, physical reactions to stress, optional panic treatment, and maintaining change). Between sessions, the participants also engaged in individually tailored physical exercises with increasing intensity. In addition to internet-based cognitive behavioral therapy sessions, participants received a brief weekly call from a clinician to provide support, encourage adherence, and provide access to the next session. Participants in the treatment-as-usual group received standard care for their noncardiac chest pain without any restrictions. Primary outcomes were cardiac anxiety, measured with the Cardiac Anxiety Questionnaire, and fear of bodily sensations, measured with the Body Sensations Questionnaire. Secondary outcomes were depression, measured using the Patient Health Questionnaire; health-related quality of life, measured using the EuroQol visual analog scale; and level of physical activity, assessed with self-report question. Additionally, a subgroup analysis of participants with depressive symptoms at baseline (PHQ-9 score ≥5) was conducted. Assessments were conducted at baseline, posttreatment, and at 3- and 12-month follow-ups. Linear mixed models were used to evaluate treatment effects. Cohen *d* was used to calculate effect sizes.

**Results:**

In the main intention-to-treat analysis at the 12-month follow-up time point, participants in the internet-based cognitive behavioral therapy group had significant improvements in cardiac anxiety (–3.4 points, 95% CI –5.7 to –1.1; *P*=.004, *d*=0.38) and a nonsignificant improvement in fear of bodily sensations (–2.7 points, 95% CI –5.6 to 0.3; *P*=.07) compared with the treatment-as-usual group. Health-related quality of life at the 12-month follow-up improved with statistical and clinical significance in the internet-based cognitive behavioral therapy group (8.8 points, 95% CI 2.8 to 14.8; *P*=.004, *d*=0.48) compared with the treatment-as-usual group. Physical activity had significantly (*P*<.001) increased during the 6-week intervention period for the internet-based cognitive behavioral therapy group. Depression significantly improved posttreatment (*P*=.003) and at the 3-month follow-up (*P*=.03), but not at the 12-month follow-up (*P*=.35). Participants with depressive symptoms at baseline seemed to have increased effect of the intervention on cardiac anxiety (*d*=0.55) and health-related quality of life (*d*=0.71) at the 12-month follow-up. In the internet-based cognitive behavioral therapy group, 84% of the participants (68/81) completed at least 5 of the 6 sessions.

**Conclusions:**

This study provides evidence that internet-based cognitive behavioral therapy with minimal therapist contact and a focus on physical activity is effective in reducing cardiac anxiety and increasing health related quality of life in patients with noncardiac chest pain.

**Trial Registration:**

ClinicalTrials.gov NCT03096925; http://clinicaltrials.gov/ct2/show/NCT03096925

## Introduction

### Background

Noncardiac chest pain is the most common final diagnosis for patients presenting with chest pain at a cardiac unit [1]. Almost half of patients with noncardiac chest pain experience ongoing complaints that negatively affect their overall quality of life after initial noncardiac chest pain diagnosis, and the associated societal costs are also high [2,3]. Typical consequences include cardiac anxiety, fear of bodily sensations, and avoidance of physical activity [2,4,5].

Psychological treatment based on cognitive behavioral therapy, delivered face-to-face by trained clinicians, has yielded promising results for noncardiac chest pain [6]. There are, however, several challenges with the dissemination of ordinary face-to-face cognitive behavioral therapy to patients with noncardiac chest pain, including a shortage of specialists who provide cognitive behavioral therapy, lack of motivation among patients with noncardiac chest pain to receive psychological treatment, and the fact that typical care providers (eg, cardiologists and cardiac nurses) often do not have the skills to provide such treatment [6].

Evidence-based face-to-face cognitive behavioral therapy for noncardiac chest pain is quite time-consuming and costly, with a normal treatment span of 6 to 12 sessions, each lasting 45 to 60 minutes each. To address this barrier, 2 randomized controlled trials [7,8] tested shorter duration face-to-face cognitive behavioral therapy specifically designed for noncardiac chest pain. For patients experiencing sustained noncardiac chest pain–related complaints 6 months after diagnosis, a 3-session face-to-face cognitive behavioral therapy with emphasis on exposure to physical activity was effective in reducing noncardiac chest pain–related complaints [7]. Furthermore, depression was a significant predictor of poor outcome [2], and fear of bodily sensations was an important mediator for change [7]. Conversely, another large randomized controlled trial [8], which included an unselected group of patients with noncardiac chest pain immediately after receiving their noncardiac chest pain diagnosis, found that 3 to 4 sessions of face-to-face cognitive behavioral therapy were not effective in reducing noncardiac chest pain–related complaints. It is difficult to fully determine the reasons for these disparate results; however, one [7] focused on physical activity and included patients who had significant complaints 6 months after the initial diagnosis of noncardiac chest pain; in contrast, the other [8] did not specifically focus on physical activity and included all patients with noncardiac chest pain immediately after their cardiac evaluation, regardless of the severity of their symptoms and duration of illness.

Internet-based cognitive behavioral therapy has the potential to increase accessibility and can be delivered at reduced costs compared with those of face-to-face cognitive behavioral therapy. Two pilot studies [9,10] have shown promising results for noncardiac chest pain: a small randomized controlled trial [9] (intervention group: n=7; treatment-as-usual control group: n=8) found that a 4-session internet-based cognitive behavioral therapy yielded greater reductions in cardiac anxiety and depression; however, because it was designed to reveal probable differences between the groups, it was underpowered to test statistical significance. Mourad et al [9] concluded that their brief internet-based cognitive behavioral therapy intervention was feasible to deliver, and though it was likely to be effective, a larger sample would be needed to adequately evaluate effectiveness. Concurrently and independently, our research group found that 6-session internet-based cognitive behavioral therapy was feasible and led to a significant reduction in cardiac anxiety for unselected noncardiac chest pain patients in an uncontrolled, open pilot study (n=10) [10]. Participants in this study were offered the internet-based cognitive behavioral therapy immediately after a cardiac condition had been excluded and received a brief weekly therapist call in addition to discuss home assignments and reinforce session content. The intervention content was based on a brief face-to-face treatment previously found to be effective in [7], and it had a similar emphasis on physical activity.

### Objectives

The purpose of this study was to further evaluate the effectiveness of the internet-based cognitive behavioral therapy intervention for noncardiac chest pain from our pilot study [10], in a randomized controlled trial with sufficient statistical power. The primary goal was to investigate the effects of the intervention on cardiac anxiety and fear of bodily sensations. The secondary goals were to evaluate changes in depression, quality of life, and physical activity, and to investigate the differential effects of this internet-based cognitive behavioral therapy in a subgroup of participants with depressive symptoms at baseline.

## Methods

### Design

This study was a 2-arm randomized controlled trial. One arm received treatment as usual, and the other received internet-based cognitive behavioral therapy for noncardiac chest pain. Participants were assessed pre- and posttreatment as well as at 3- and 12-month follow-up time points.

### Participants and Recruitment

Participants were recruited at the Sørlandet Hospital, Kristiansand, Norway. A member of the research group screened all patients with chest pain as their main complaint who had been referred to the cardiac unit (including an inpatient and an outpatient unit) for participation. The member of the research group informed cardiologists about possible eligible patients. The cardiologists used a checklist with inclusion and exclusion criteria as well as the definition of noncardiac chest pain to determine the eligibility of each patient. The cardiologist briefly explained the project to eligible patients. If the patient agreed, a member of the research group immediately provided further verbal and written information about the trial before the recruitment procedure.

Eligible patients were aged 18 to 70 years, had no cardiac or other somatic disease that could explain their chest pain symptoms, no history of or ongoing severe heart disease, and did not meet any of the following exclusion criteria: (1) language difficulties; (2) inability to perform at least moderate physical activity due to physical constraints; (3) obvious cognitive impairment (eg, severe intellectual disability, psychosis, dementia, or intoxication); (4) no regular access to a computer or tablet with internet connection; and (5) severe somatic comorbidities (eg, cancer, severe kidney failure).

If patients had been examined with coronary computed tomography angiography, only patients with coronary artery blockage less than 50% were eligible. All eligible patients were asked to sign a paper consent form if they agreed to participate.

### Intervention

**Figure 1:**
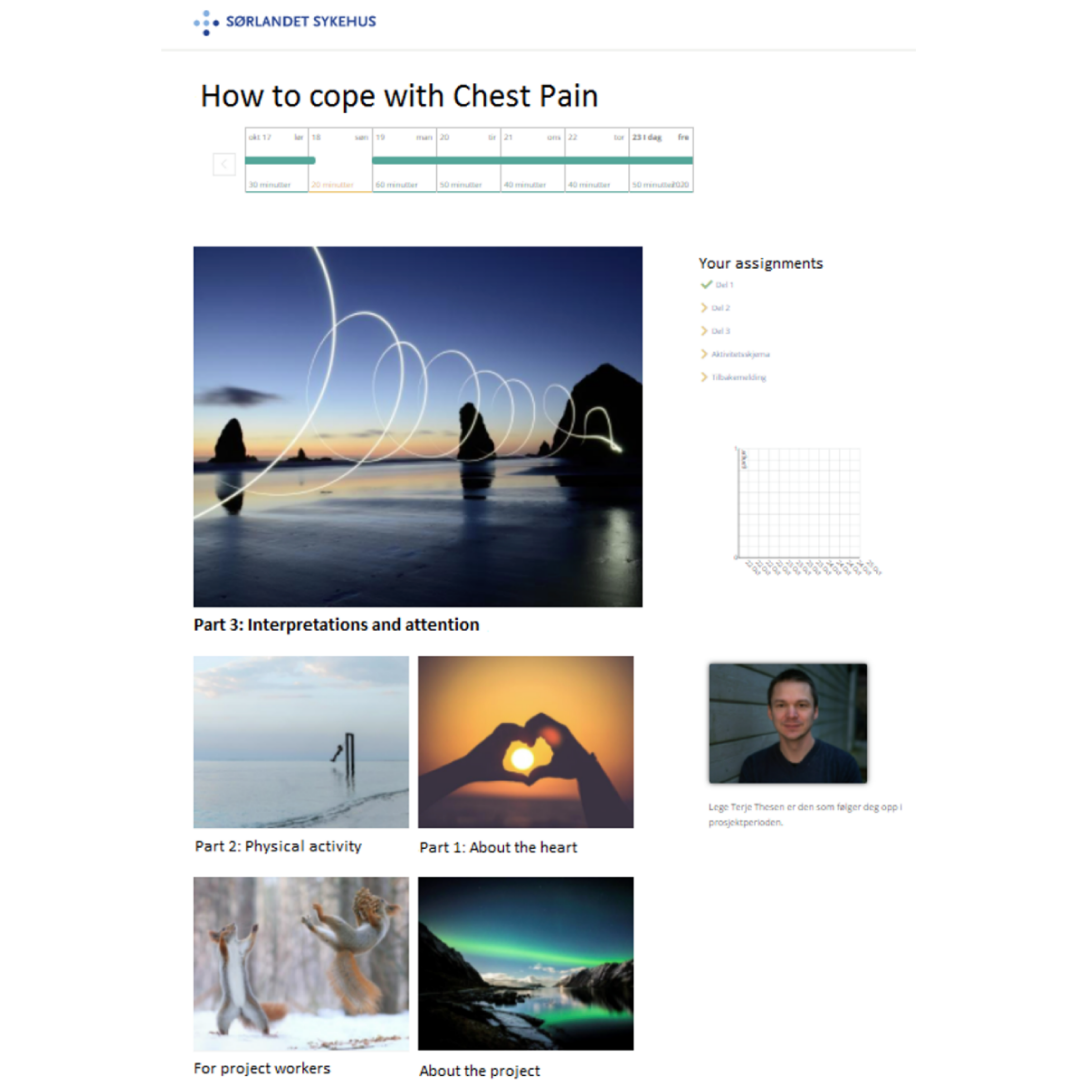
Screenshot from the internet-based cognitive behavioral therapy at week 3.

The brief internet-based cognitive behavioral therapy with minimal therapist contact has been previously described [10]. The intervention was adjusted and improved based on feedback from participants in the pilot study [10]. Internet-based cognitive behavioral therapy sessions (Figure 1; Multimedia Appendix 1) were completed autonomously once per week for 6 weeks. Between sessions, participants received support calls from a therapist.

The internet-based cognitive behavioral therapy was specifically designed and adapted for noncardiac chest pain (1) to provide an alternative explanation for their chest pain, (2) to provide tools and education on how to handle bodily discomfort, (3) to encourage physical activity with the aim that the participants experienced its safety, and (4) to provide information on strategies that can prevent relapse.

**Figure 2:**
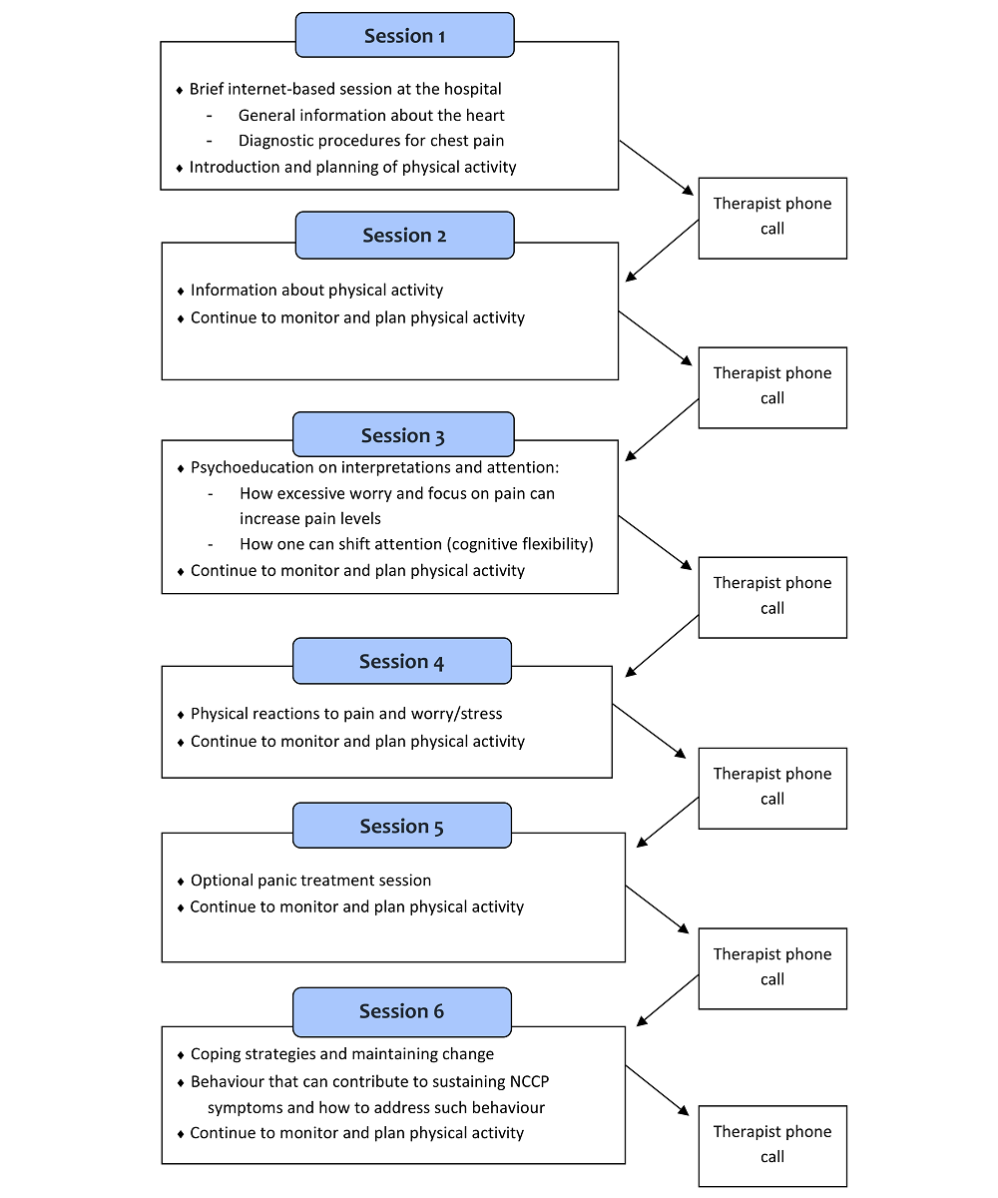
Flowchart for internet-based cognitive behavioral therapy. NCCP: noncardiac chest pain [10].

The first session was completed on a computer or tablet at the cardiac unit immediately after the cardiac examination was completed. The sessions covered different topics relevant to the noncardiac chest pain patients (Figure 2). At the end of every session, each participant completed a detailed activity plan for the following week. Adherence to these activities was reported to the therapist electronically. The activity plan focused on individually tailored physical exercises with increasing intensity. In addition, participants were encouraged to perform an attention task regularly after the third session. Once a week, a brief support call (5-7 minutes duration), designed to reinforce the session content, discuss home-based tasks, and troubleshoot problems, was made by the same therapist. The support call had 3 predefined elements: (1) discussion about the previous week’s physical activities and tasks, (2) discussion about the previous week’s session, and (3) general information about the following week’s session. The participants were given access to the subsequent week’s internet-based cognitive behavioral therapy session after the support call. The completed sessions remained accessible to the participant throughout the project period.

Participants in the control group received treatment as usual—personal consultation with a doctor at the cardiac unit after completing the cardiologic investigations. The consultation included information regarding the results of the examination and advice on medication, diet, and physical activity. The intervention group received internet-based cognitive behavioral therapy in addition to personal consultation at the cardiac unit. Lottery tickets (each with a price of €5, approximately US $5.67) were sent to all participants after they completed assessments to enhance adherence.

A unique code was used to access the intervention platform. The intervention platform included session-by-session content and homework exercises. Data were stored using pseudonyms on a secure server in Oslo, Norway. This server is backed up every 24 hours by another secure server in Amsterdam, Netherlands. Only the therapist could link the code to the participant. No personal identifiable information was collected, and IP addresses were not logged.

### Assessments and Outcomes

Participants completed web-based self-report questionnaires at baseline and posttreatment, and at 3- and 12-month follow-up time points. The primary outcome measures were sum scores for the 18-item Cardiac Anxiety Questionnaire (CAQ), which measures worry or fear of heart sensations, avoidance of physical activity due to fear of eliciting heart symptoms, heart-focused attention, and reassurance seeking [11,12], and the 17-item Body Sensations Questionnaire (BSQ), which measures worry about specific bodily sensations [13].

The CAQ has a sum score that ranges from 0 to 72, with each item scored on a scale of 0 to 4. Higher scores indicate more symptoms. The CAQ is well validated, has good internal consistency (Cronbach α=.84), and high test–retest reliability (*r*=0.88) [14].

The BSQ has a total score that ranges from 17 to 85, with each item scored on a scale of 1 (not worried) to 5 (extremely frightened) [15]. The BSQ has been used in several noncardiac chest pain trials [4,7], has high internal consistency (Cronbach α=.87), and moderate test–retest reliability (*r*=0.66) [13].

Secondary outcome measures were the 9-item Patient Health Questionnaire (PHQ-9) score, the EuroQol Visual Analog Scale (EQ-VAS) score, and physical activity assessment.

The PHQ-9 includes and assesses level of depression. Each item is scored from 0 to 3, and the sum range ranges from 0 to 27. Higher scores indicate greater depression. We used a sum score ≥5 to indicate mild depressive symptoms. The PHQ-9 is well validated, has high internal consistency (Cronbach α=.89), high test–retest reliability (*r*=0.84) [16], and also validated in a web-based format [17].

The EQ-VAS measures the user’s overall rating of their health [18] using a visual analog scale that is presented as a line from 0 to 100, where 0 is defined as the worst imaginable health state and 100 is the best.

Participants were further assessed using an investigator-developed, nonvalidated question that assessed physical activity level: “How many times each week on average do you perform physical activity more than 30 min?”.

### Clinical Relevance

No minimal clinically important difference thresholds have been established for the CAQ and BSQ. For PHQ-9, we defined a difference of 3 points or a reduction of 20% from baseline as the minimal clinically important difference based on values from studies [19-21]. The minimal clinically important difference for EQ-VAS has not been established specifically for noncardiac chest pain, but previous studies on cancer and COPD [22-24] have proposed that change in the range of 5.4-7.0 points is clinically relevant.

### Adverse Events

The risk of serious adverse events due to internet-based cognitive behavioral therapy was considered low prior to the trial, and adverse events were not systematically collected. PHQ-9 scores were reviewed for each participant. Participants with a score ≥20 (severe depression) or a score of 3 on question 9 (which addresses suicidal ideation) were contacted. A decision by the principal investigator (TT) was made regarding the need for a psychiatric evaluation and whether to contact their general practitioner.

### Randomization and Blinding

The participants were randomized 1:1 without stratification or the use of known blocks. The study used a web-based randomization procedure performed at a remote location (Web-CRF at the Norwegian University of Science and Technology in Trondheim). Because the intervention was internet-based cognitive behavioral therapy, blinding was not possible for the participant or therapist. Outcome measures were collected electronically, and except for the PHQ-9 scores, were not known to the therapist or the research group during the study period.

### Sample Size

The study was powered to detect a Cohen *d* effect size of at least 0.5. The power calculations were based on the results from the pilot trial [10] and a previous study with brief face-to-face cognitive behavioral therapy for noncardiac chest pain [7]. A 2-sided test, with α<.05 and power >80% was used, and a sample size of 63 participants in each group was needed to detect a mean difference between groups of 5.1 points on the BSQ with a standard deviation of 10.2. To tolerate an anticipated 20% dropout rate, a total sample size of 80 participants in each group was deemed appropriate.

### Data Analysis

Primary analyses were conducted using intention-to-treat analysis, in which participants were analyzed according to the group they had been randomized. In the secondary per-protocol analysis, participants were excluded if they (1) did not complete at least 5 of 6 sessions; (2) did not complete all assessments; or (3) were found after randomization not to have been eligible based on the inclusion and exclusion criteria. Linear mixed models were used to evaluate treatment effects. Linear mixed models take into account repeated measures for each participant. The fixed part of the model included a group indicator variable (intervention or treatment as usual), a time variable (posttreatment, 3-month follow-up, and 12-month follow-up), and an interaction term between the group indicator and time variable. Baseline scores were included as covariates in the fixed portion of the analysis. Maximum likelihood estimations were used to estimate the effects on all outcomes. Between-group effect sizes (Cohen *d*) were calculated for selected time points. We used SPSS statistical software (version 25, IBM Corp) for all analyses.

### Patient Involvement

One person with noncardiac chest pain experience participated in the development of the internet-based cognitive behavioral therapy intervention. This person reviewed each session during the development phase and provided input on the relevance of the topics and the didactics of the intervention.

### Ethics

The study protocol was approved by the Regional Committee for Medical Research Ethics (2014/2031).

## Results

**Figure 3:**
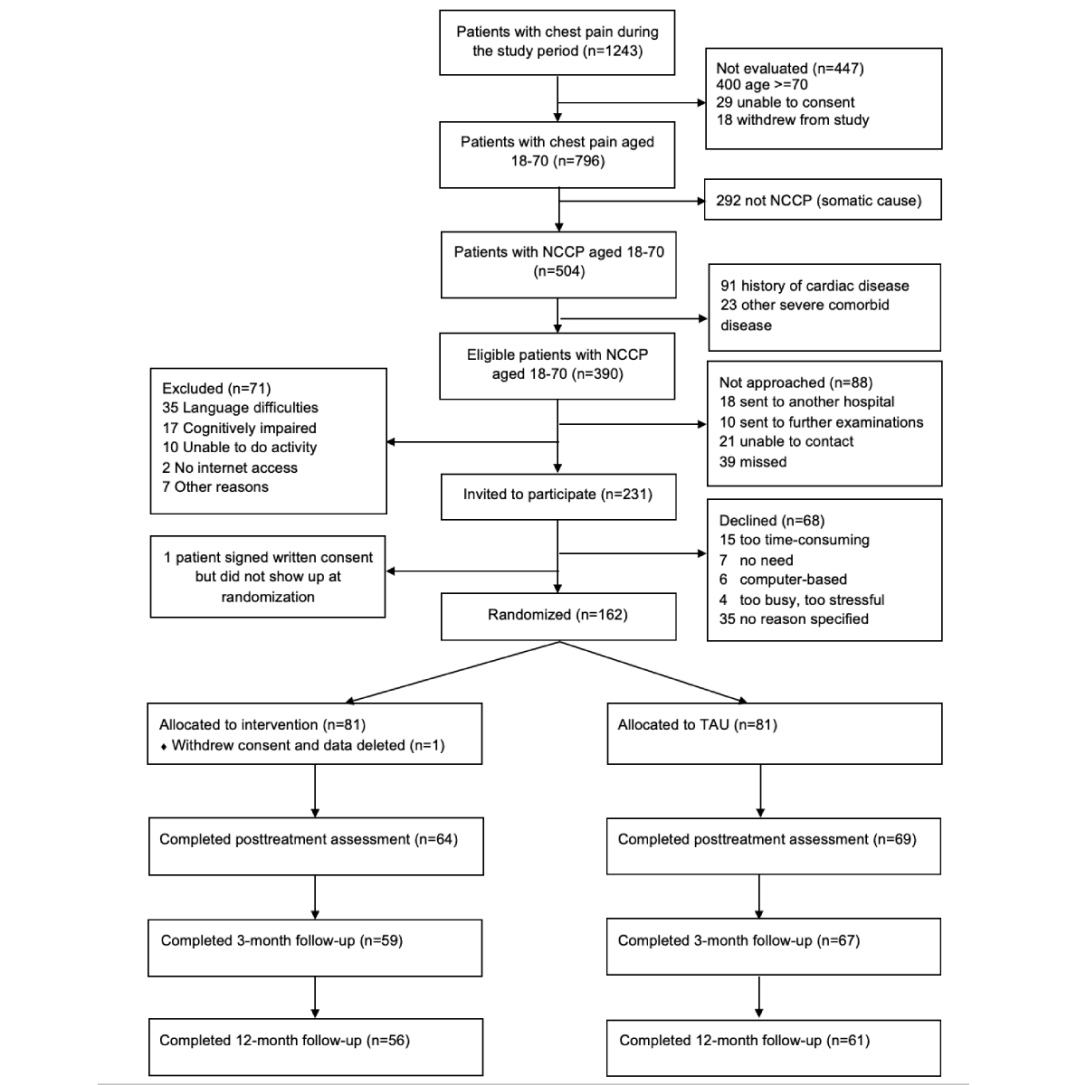
Flow of participants during the study period. NCCP: non-cardiac chest pain; TAU: treatment as usual.

Participants were recruited consecutively (Figure 3) from April 3, 2017 to March 26, 2018, with a 6-week pause from May to June 2017 (total recruitment period: 10.5 months).

Of 796 patients with chest pain who were screened for participation, 504 patients fulfilled criteria for noncardiac chest pain; 231 patients were invited to participate, and 162 accepted and were randomly allocated (intervention group: n=81; treatment-as-usual group: n=81). One participant withdrew consent and requested that their collected data be deleted; therefore, 161 participants were included in the intention-to-treat analysis (Table 1). One participant had severe comorbid disease, which was discovered after randomization, and another had severe depressive symptoms that required specialist treatment; these patients were excluded from the per-protocol analysis. In the intervention group, 68 (84%) completed the internet-based cognitive behavioral therapy (at least 5 of 6 sessions). Posttreatment assessments were completed for 133, 3-month assessments for 126, and 12-month assessments for 117 participants. In the per-protocol analysis, 55 participants in the intervention group and 61 participants in the treatment-as-usual group were included. The groups were well balanced for demographic and clinical data.

**Table 1:** Demographic and clinical data collected at baseline.

Characteristic			Intervention group (n=80), n (%)		Treatment as usual (n=81), n (%)		Full sample (n=161), n (%)
**Gender**							
	Men	36 (45)		38 (47)		74 (46)	
	Women	44 (55)		43 (53)		87 (54)	
Age (years), median (range)			53 (20-69)		51 (30-69)		52 (20-69)
Married or cohabiting			64 (80)		68 (84)		132 (82)
**Highest education**							
	Primary or high school	18 (23)		18 (22)		36 (22)	
	Vocational school	29 (36)		30 (37)		59 (37)	
	College or university	33 (41)		33 (41)		66 (41)	
**Occupational status**							
	Full work	43 (54)		48 (59)		91 (57)	
	Part time work	16 (20)		9 (11)		25 (16)	
	Disability	14 (18)		18 (22)		32 (20)	
	Retired	4 (5)		5 (6)		9 (6)	
	Sick leave	3 (4)		1 (1)		4 (3)	
**Comorbid disorders^a^**							
	Somatic	42 (53)		41 (51)		82 (52)	
	Mental health	10 (13)		11 (14)		21 (13)	
**Chest pain duration**							
	0-1 month	22 (28)		24 (30)		46 (29)	
	2-6 months	29 (36)		28 (35)		57 (35)	
	>6 months	29 (36)		29 (36)		58 (36)	
**Chest pain frequency**							
	Never	2 (3)		4 (5)		6 (4)	
	Seldom	41 (51)		37 (46)		78 (48)	
	Weekly	20 (25)		26 (32)		46 (29)	
	Daily	17 (21)		14 (17)		31 (19)	

^a^According to self-report.

### Primary Outcomes

In the intention-to-treat analysis (Table 2), statistically significant reductions in CAQ score at the 3-month (*P*=.03) and 12-month follow-up (*P*=.004) were observed in the intervention group compared with the treatment-as-usual group. The effect size at 12-month follow-up was small to moderate (*d*=0.38). Accordingly, the BSQ scores were significantly reduced at posttreatment (*P*=.03) and at the 3-month follow-up (*P*=.04). 

In the secondary per-protocol analysis (Table 2), participants in the intervention group showed statistically significant reductions in CAQ scores at the 3-month (*P*=.02) and 12-month (*P*=.002) follow-up compared with those in the treatment-as-usual group. Hence, the BSQ scores in the per-protocol analysis were significantly reduced at all time points (posttreatment: *P*=.02; 3-month follow-up: *P*=.008; 12-month follow-up: *P*=.04).

**Table 2:** Comparison of outcome measures between groups.

Outcome				Score, mean (SD)				Intention-to-treat analysis				Per-protocol analysis		
				Intervention^a^		Treatment as usual^b^		95% CI		*P* value^c^		95% CI		*P* value^d^
**Primary**														
	**Cardiac Anxiety Questionnaire**													
		Baseline	23.2 (9.8)		23.4 (10.3)		Reference		N/A^e^		Reference		N/A	
		Post	17.5 (9.1)		18.4 (9.7)		–1.0 (–3.3 to 1.2)		.36		–1.2 (–3.6 to 1.1)		.31	
		3 months	14.5 (7.9)		18.1 (10.4)		–2.5 (–4.8 to –0.2)		.03		–2.9 (–5.3 to –0.5)		.02	
		12 months	13.8 (8.7)		17.6 (11.1)		–3.4 (–5.7 to –1.1)		.004		–3.7 (–6.0 to –1.3)		.002	
	**Body Sensations Questionnaire**													
		Baseline	36.8 (11.6)		36.7 (12.9)		Reference		N/A		Reference		N/A	
		Post	31.3 (9.7)		33.5 (12.5)		–3.1 (–6.0 to –0.3)		.03		–3.5 (–6.5 to –0.5)		.02	
		3 months	29.7 (9.9)		32.2 (12.6)		–3.0 (–5.9 to –0.1)		.04		–4.1 (–7.1 to –1.1)		.008	
		12 months	29.2 (10.3)		31.3 (12.2)		–2.7 (–5.6 to 0.3)		.07		–3.1 (–6.1 to –0.1)		.04	
**Secondary**														
	**Patient Health Questionnaire–9**													
		Baseline	6.9 (4.7)		6.9 (5.0)		Reference		N/A		Reference		N/A	
		Post	4.9 (3.9)		6.6 (5.2)		–1.6 (–2.7 to –0.6)		.003		–2.1 (–3.2 to –0.9)		<.001	
		3 months	4.4 (3.5)		5.9 (4.6)		–1.3 (–2.4 to –0.2)		.03		–1.7 (–2.8 to –0.5)		.004	
		12 months	4.8 (3.8)		5.3 (5.0)		–0.5 (–1.7 to 0.6)		.35		–0.9 (–2.0 to 0.3)		.13	
	**EuroQol Visual Analog Scale**													
		Baseline	63.5 (20.2)		63.4 (19.4)		Reference		N/A		Reference		N/A	
		Post	69.0 (19.9)		62.0 (21.6)		7.7 (1.9 to 13.4)		.009		10.5 (4.4 to 16.6)		.001	
		3 months	71.8 (19.0)		61.1 (20.6)		10.3 (4.5 to 16.2)		.001		12.1 (6.1 to 18.2)		<.001	
		12 months	71.8 (19.0)		61.8 (22.4)		8.8 (2.8 to 14.8)		.004		10.0 (4.0 to 16.1)		.001	
	**Physical activity^f^**													
		Baseline	3.0 (1.9)		3.4 (2.6)		Reference		N/A		Reference		N/A	
		Post	4.5 (1.9)		3.3 (2.1)		1.3 (0.8 to 1.8)		<.001		1.6 (1.1 to 2.1)		<.001	
		3 months	3.7 (1.9)		3.4 (2.4)		0.3 (–0.2 to 0.8)		.19		0.5 (0 to 1.0)		.06	
		12 months	3.7 (2.0)		3.5 (2.1)		0.1 (–0.4 to 0.6)		.65		0.3 (–0.2 to 0.7)		.30	

^a^Intervention: baseline (n=80); post (n=64); 3 months (n=59); 12 months (n=56).

^b^Treatment as usual: baseline (n=81); post (n=69); 3 months (n=67); 12 months (n=61).

^c^Estimated mean differences between groups adjusted for baseline values (linear mixed models).

^d^Estimated mean differences between groups adjusted for baseline values (linear mixed models); n=55 in the intervention group and n=61 in the treatment-as-usual group.

^e^N/A: not applicable.

^f^Number of times with moderate physical activity >30 minutes a week.

### Secondary Outcomes

In the intention-to-treat analysis, participants in the intervention group reported a statistically significant improvement in depression posttreatment (*P*=.003) and at the 3-month follow-up (*P*=.03) compared with the treatment-as-usual group.

Health-related quality of life improved, reaching both statistical (posttreatment: *P*=.009; 3-month follow-up: *P*=.001; 12-month follow-up: *P*=.004) and clinical significance in the intervention group at all time points. The effect size at 12-month follow-up was small to moderate (*d*=0.48). Physical activity increased significantly in the intervention group posttreatment compared with treatment-as-usual group (*P*<.001). In the per-protocol analyses, all secondary measures improved to a greater extent in the intervention group than in the treatment-as-usual group (Table 2).

Participants scoring ≥5 on the PHQ-9 showed greater improvements in all outcome measures in favor of the intervention group in the intention-to-treat analysis and also in the per-protocol analysis (except for cardiac anxiety at posttreatment) (Table 3). Effect sizes were moderate for CAQ (*d*=0.55) and EQ-VAS (*d*=0.71) scores at the 12-month follow-up.

**Table 3:** Comparison of outcome measures for participants with baseline depression scores (PHQ-9 ≥5).

Outcome			Score, mean (SD)			Intention-to-treat analysis			Per-protocol analysis	
			Intervention^a^	Treatment as usual^b^	95% CI		*P* value^c^	95% CI		*P* value^d^
**Primary**										
	**Cardiac Anxiety Questionnaire**									
		Baseline	25.6 (9.9)	25.6 (10.3)	Reference		N/A^e^	Reference		N/A
		Post	20.2 (9.6)	20.7 (10.0)	–0.6 (–3.6 to 2.3)		.66	–0.6 (–3.8 to 2.6)		.71
		3 months	16.3 (8.4)	21.8 (10.7)	–3.9 (–6.9 to –0.9)		.01	–4.6 (–7.8 to –1.4)		.006
		12 months	15.3 (9.4)	21.3 (12.4)	–4.7 (–7.9 to –1.6)		.003	–5.0 (–8.3 to –1.8)		.002
	**Body Sensations Questionnaire**									
		Baseline	39.7 (12.0)	38.6 (14.2)	Reference		N/A	Reference		N/A
		Post	32.2 (10.3)	34.0 (13.0)	–3.7 (–7.4 to –0.1)		.04	–4.1 (–8.1 to –0.1)		.046
		3 months	30.3 (10.9)	33.0 (12.6)	–4.0 (–7.8 to –0.3)		.04	–5.7 (–9.6 to –1.7)		.006
		12 months	31.4 (11.4)	33.1 (14.0)	–3.5 (–7.4 to 0.4)		.08	–4.1 (–8.0 to –0.1)		.04
**Secondary**										
	**Patient Health Questionnaire–9**									
		Baseline	9.4 (4.1)	9.3 (4.4)	Reference		N/A	Reference		N/A
		Post	6.4 (4.1)	9.1 (5.0)	–2.4 (–4.0 to –0.8)		.003	–3.1 (–4.8 to –1.4)		.001
		3 months	5.6 (3.8)	7.8 (4.4)	–1.7 (–3.3 to –0.0)		.047	–2.3 (–4.1 to –0.6)		.008
		12 months	5.9 (4.1)	7.7 (5.3)	–1.3 (–3.0 to 0.4)		.14	–1.8 (–3.5 to –0.1)		.04
	**EuroQol Visual Analog Scale**									
		Baseline	59.9 (21.9)	58.3 (20.5)	Reference		N/A	Reference		N/A
		Post	65.9 (21.0)	55.2 (21.0)	9.9 (2.1 to 17.7)		.01	14.4 (5.7 to 23.1)		.001
		3 months	68.0 (20.4)	54.3 (20.9)	11.3 (3.2 to 19.4)		.007	13.5 (4.7 to 22.2)		.003
		12 months	70.9 (21.0)	54.6 (24.8)	12.4 (4.1 to 20.8)		.004	14.2 (5.4 to 22.9)		.002
	**Physical activity^f^**									
		Baseline	2.8 (1.7)	3.2 (2.3)	Reference		N/A	Reference		N/A
		Post	4.5 (2.0)	3.4 (2.2)	1.3 (0.7 to 2.0)		<.001	1.7 (1.1 to 2.4)		<.001
		3 months	3.7 (2.0)	3.4 (2.3)	0.4 (–0.3 to 1.1)		.24	0.6 (–0.1 to 1.2)		.09
		12 months	3.7 (2.1)	3.4 (2.3)	0.2 (–0.4 to 0.9)		.48	0.4 (–0.2 to 1.1)		.20

^a^Intervention: baseline (n=51); post (n=40); 3 months (n=35); 12 months (n=34).

^b^Treatment as usual: baseline (n=53); post (n=43); 3 months (n=41); 12 months (n=35).

^c^Estimated mean differences between groups adjusted for baseline values (linear mixed models).

^d^Estimated mean differences between groups adjusted for baseline values (linear mixed models); n=34 in the intervention group and n=35 in the treatment-as-usual group.

^e^N/A: not applicable.

^f^Number of times with moderate physical activity >30 minutes a week.

### Adverse Events and Protocol Deviations

The participants of the intervention group did not report any serious adverse events in the telephone contacts with the therapist. Two participants screened had serious depression (scores >20 on the PHQ-9): one patient in the treatment-as-usual group required an acute psychiatric evaluation and was referred to the psychiatric specialist team. The other participant (in the intervention group) wanted to continue with the intervention. In agreement with the participant, the general practitioner was informed of the intervention, and the participant continued the treatment.

## Discussion

### Principal Findings

In this randomized controlled trial, we compared the effectiveness of internet-based cognitive behavioral therapy and treatment as usual in patients with noncardiac chest pain. Our findings provide strong evidence for the effectiveness of internet-based cognitive behavioral therapy after a negative cardiac evaluation in a hospital setting. Participants receiving internet-based cognitive behavioral therapy experienced improvements in cardiac anxiety, fear of bodily sensations, depressive symptoms, health-related quality of life, and physical activity level. Participants with depressive symptoms at baseline showed greater improvements after internet-based cognitive behavioral therapy on almost all outcome measures compared with those who were not depressed at baseline.

Most (163/231, 70%) patients who were invited agreed to participate, and 84% of participants (68/81) in the intervention group completed at least 5 of the 6 internet-based cognitive behavioral therapy sessions. This indicates that the intervention had a high acceptability. The cumulative therapist time spent for each internet-based cognitive behavioral therapy participant totaled 60 to 70 minutes, including the time spent providing information about the intervention at the hospital. This makes the intervention considerably less costly and time-consuming (per patient) than even brief face-to-face cognitive behavioral therapy.

### Primary Outcomes

Cardiac anxiety did not significantly improve at the posttreatment assessment in the internet-based cognitive behavioral therapy group compared with the treatment-as-usual group. However, although the improvements within the intervention group during the intervention period were large, improvements in the treatment-as-usual group were also substantial. This may indicate that an examination by a cardiologist, which is often combined with computed tomography angiography, can contribute to reducing cardiac anxiety. One might expect that improvement in cardiac anxiety in the treatment-as-usual group would be temporary, but the improvements were similarly maintained in the treatment-as-usual group at the 3- and 12-month follow-ups. However, cardiac anxiety in the intervention group improved significantly more than in the treatment-as-usual group at the 3- and 12-month follow-ups in both the intention-to-treat and per-protocol analyses, suggesting that the exclusion of cardiac conditions combined with internet-based cognitive behavioral therapy yield a better effect on cardiac anxiety over time.

As with cardiac anxiety, the improvements in fear of bodily sensations within the intervention group were large and comparable to findings in a previous study [7]. However, the treatment-as-usual group also improved substantially; at the 12-month follow-up, the intervention group reported a nonsignificant improvement of 2.7 points (*P*=.07) in the intention-to-treat analysis and a statistically significant improvement of 3.1 points (*P*=.04) in the per-protocol analysis. These improvements in the treatment-as-usual group were somewhat surprising, given that in [7], the treatment-as-usual group actually showed increased BSQ scores at the 12-month follow-up. In [7], the intervention group had substantially and significantly less symptoms on BSQ at 12 months (between-group difference: 7.5 points). The smaller improvement in BSQ in our study might be explained by the fact that the participants in [7] had significant noncardiac chest pain-related complaints at the time of study inclusion (6 months after receiving a noncardiac chest pain diagnosis). Therefore, in [7], the time period most likely associated with a naturalistic remission of noncardiac chest pain symptoms was completed, thus eliminating potential participants with very mild symptoms and little room for improvement from the analysis of key outcome measures.

### Secondary Outcomes

PHQ-9 depression scores improved significantly more in the intervention group than in the treatment-as-usual group posttreatment (*P*=.003) and at the 3-month follow-up (*P*=.03), but there was no significant difference at the 12-month follow-up (*P*=.35). It is important to note that the sample had a low baseline score of 6.9 on the PHQ-9, indicating only mild depressive symptoms on average. These low baseline scores reduced the likelihood of finding a significant difference in depression between the internet-based cognitive behavioral therapy and treatment-as-usual groups. Health-related quality of life improved substantially in the intervention group, whereas a slight deterioration was observed in the treatment-as-usual group. This may suggest that even if patients with noncardiac chest pain experience improvement in cardiac anxiety and fear of bodily sensations without specific treatment, they might struggle to regain a normal quality of life. This corresponds well with the findings of other studies [5,25,26]. The improvement in EQ-VAS in the intervention group was statistically significant (posttreatment: *P*=.009; 3 months: *P*=.001; 12 months: *P*=.004) and clinically relevant at all points of time.

There was a significant increase in physical activity in the intervention group posttreatment (*P*<.001), suggesting that the participants adhered to the physical activity–related elements of our intervention. This initial increase in physical activity in the internet-based cognitive behavioral therapy group was not maintained at the 3- and 12-month follow-up. Clinical impressions suggest that a key element in the internet-based cognitive behavioral therapy was exposure to increased heart rates, followed by the observation that the participants did not experience a health catastrophe. We did not include a specific measure to monitor the intensity of physical activities, but participants who were more physically active also exposed themselves more often to activities that increased their heart rate. We hypothesize that even a temporary increase in activity level with a focus on achieving a high heart rate may have lasting positive effects on cardiac-related anxiety and psychological limitations associated with physical activity.

### Subgroup Analysis of Patients With a PHQ-9 Score >5 at Baseline

Studies that included noncardiac chest pain patients regardless of baseline noncardiac chest pain–related symptom levels, such as [8] and our study, might have difficulty detecting significant differences when baseline scores are low. We found larger effects on most outcome measures among internet-based cognitive behavioral therapy participants with baseline PHQ-9 scores ≥5. Patients with noncardiac chest pain and with depressive symptoms might benefit more from internet-based cognitive behavioral therapy, and PHQ-9 scores should be further analyzed as a possible predictor of treatment effects. Given that internet-based cognitive behavioral therapy was effective for the participant group as a whole, offering a low-cost intervention with no known side effects to all patients with noncardiac chest pain regardless of symptom levels at a busy cardiac unit might be advantageous from an implementation perspective, even though internet-based cognitive behavioral therapy might not be needed for those with low depression scores at baseline.

### Comparison With Prior Work

Previously, only one small pilot randomized controlled trial [9] tested the effectiveness of internet-based cognitive behavioral therapy for noncardiac chest pain and did not find statistically significant differences in CAQ or BSQ scores; however, the study was clearly underpowered. In another recent study [8], 3- or 4-session face-to-face cognitive behavioral therapy were compared with treatment as usual in an unselected group of 424 noncardiac chest pain patients recruited at the hospital immediately after their cardiac examination, and health anxiety improved at the 3-month follow-up in the intervention group compared with in the treatment-as-usual group, but these improvements were not sustained at the 12-month follow-up. The main difference between [8] and our study is that our intervention focused on exposure to physical activity (which was not emphasized in [8]). Notably, Jonsbu et al [7] found sustained improvements in BSQ, avoidance of physical activity and depression 12 months after the participants completed their exercise-focused cognitive behavioral therapy treatment. Our focus on physical activity may account for the enduring effects of our intervention. Cognitive behavioral therapy is often more powerful when maladaptive cognitions are challenged during systematic exposure [27].

### Strengths and Limitations

This study has several significant strengths. First, a high percentage of potential candidates accepted and participated (162/231, 70%), perhaps because the patients were recruited in a cardiac setting. Second, adherence to the internet-based cognitive behavioral therapy was high (68/81, 84%), and sample retention at the 12-month follow-up was acceptable (117/162, 72.2%). Third, the study was performed in a routine clinical setting with few exclusion criteria, which strengthens the generalizability of the results. Fourth, the intervention required approximately 1 hour of therapist time per patient and was easy to administer. Finally, outcome measures were collected electronically, and randomization was performed electronically at a remote location.

This trial also had some limitations. First, the therapist (TT) who conducted all the check-in calls during this study participated in the development of the intervention and had previous cognitive behavioral therapy training, which may have increased the effect of internet-based cognitive behavioral therapy in this study. In addition, all check-in calls were made by the same therapist, except during a period of a few weeks. Second, the control group only received treatment as usual (we did not use a sham intervention as a control condition). Third, neither the therapist nor the participants could be blinded to the intervention. Fourth, computer literacy and internet access were required in order to utilize the intervention. Finally, the assessment of physical activity and analysis of PHQ-9 ≥5 were not described in the original trial registration. A recent study [8] found that brief cognitive behavioral therapy without a particular focus on physical activity does not have an effect on noncardiac chest pain, suggesting that physical activity is an important aspect of the treatment. The analysis of PHQ-9 ≥5 was added because previous research found that higher depression scores predicted poorer outcomes in patients with noncardiac chest pain [2,28].

### Clinical Implications and Further Research

An easily accessible, low-cost, and effective internet-based cognitive behavioral therapy for noncardiac chest pain has the potential to improve care and quality of life in a large and underserved group of patients. Further research is needed to investigate whether standard staff working at the cardiac unit can provide this type of care effectively and to determine whether certain patient subgroups benefit more or less from this type of intervention.

### Conclusions

This study provides evidence that internet-based cognitive behavioral therapy with minimal therapist contact and with a focus on physical activity is effective in reducing cardiac anxiety and increasing health-related quality of life in patients with noncardiac chest pain.

## Appendix

Multimedia Appendix 1 Snapshots from the intervention.

Multimedia Appendix 2 CONSORT-EHEALTH checklist (V 1.6.1).

## Abbreviations

BSQ: Body Sensations Questionnaire
CAQ: Cardiac Anxiety Questionnaire
EQ-VAS: EuroQol Visual Analog Scale
PHQ-9: Patient Health Questionnaire–9
