# Supplementary material for: Effectiveness of Internet-Based Cognitive Behavioral Therapy With Telephone Support for Noncardiac Chest Pain: Randomized Controlled Trial
Source: J Med Internet Res. 2022 Jan 24;24(1):e33631. doi: 10.2196/33631 (PMC8822426; doi:10.2196/33631)

**Snapsshots from the intervention**

**Snapshot 1:** Log in page – the participants enters a unique code to access the intervention at the webpage www.brystsmerter.no


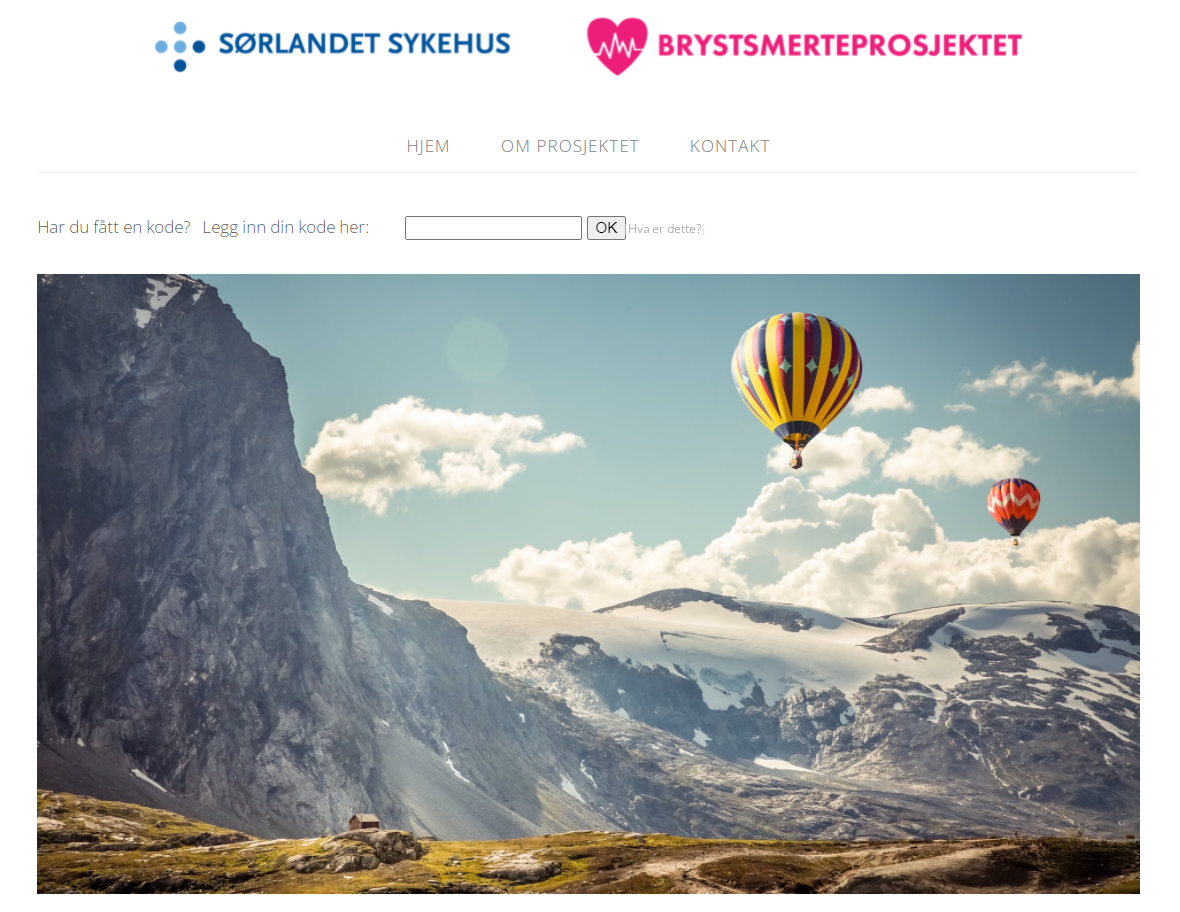


**Snapshot 2:** Dashbord when the participant log in, in this snapshot the participant is at session 3.


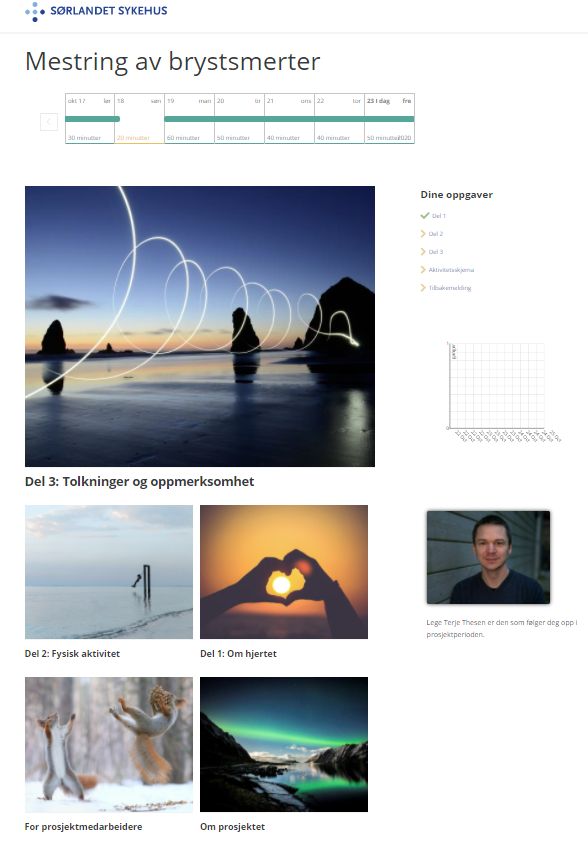


**Snapshot 3:** With explanations to some of the features in the dashboard


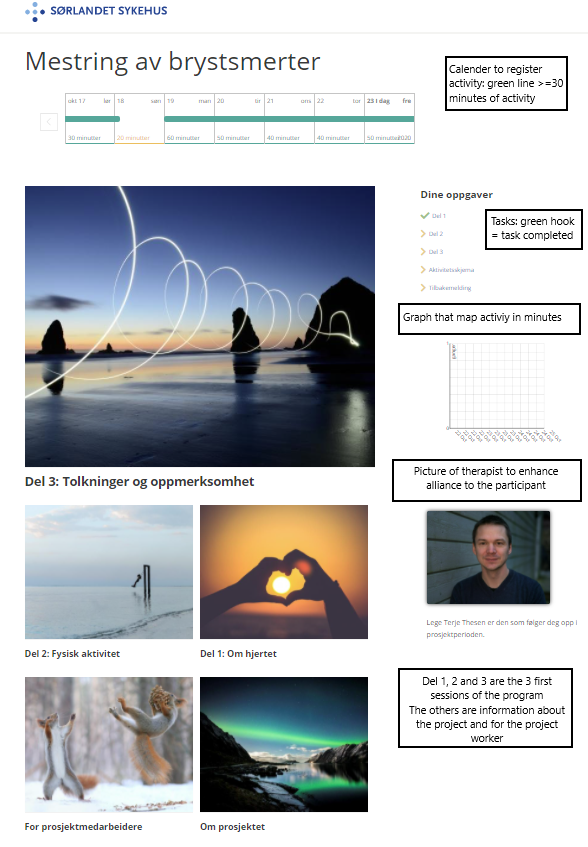


**Snapshot 4:** Inside session 3
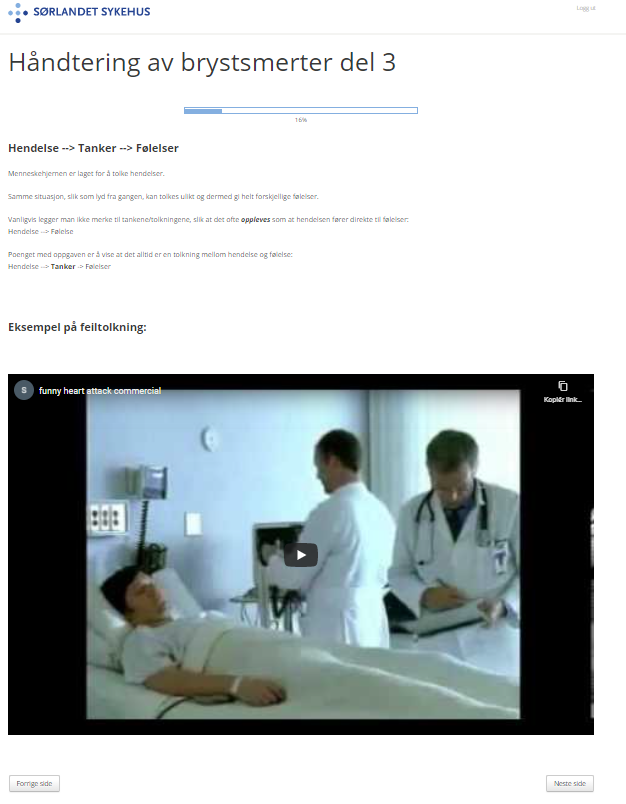


**Snapshot 5:** Inside session 3, about how intrepretations affect feelings and a small task/experiment related to the topic.


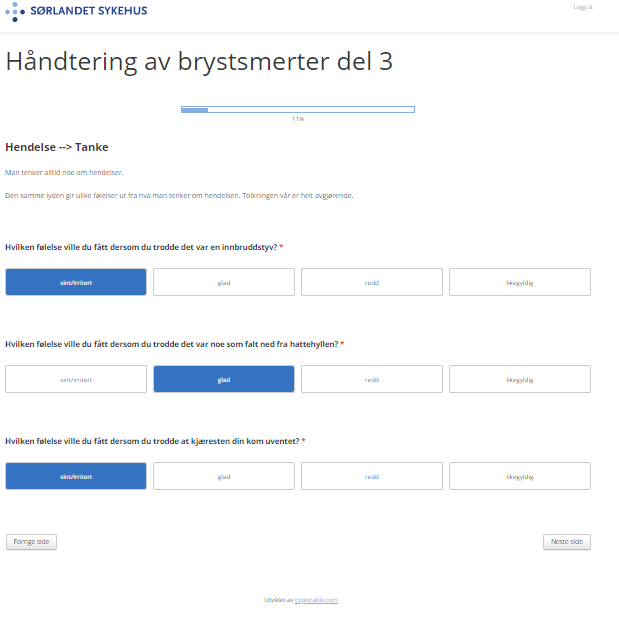


**Snapshot 6:** Inside session 5, about bodily reactions related to stress/panic
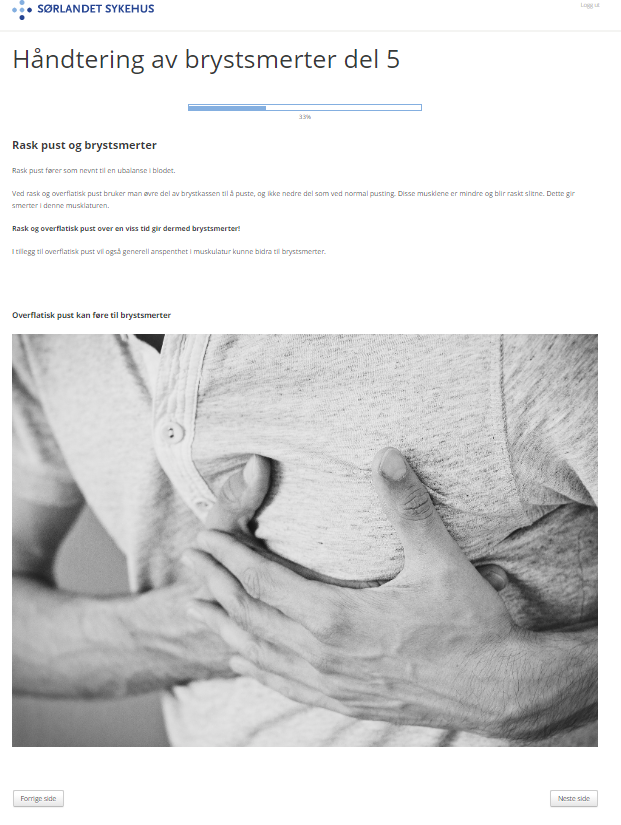


**Snapshot 7:** Inside session 6, about negative effects of avoidance
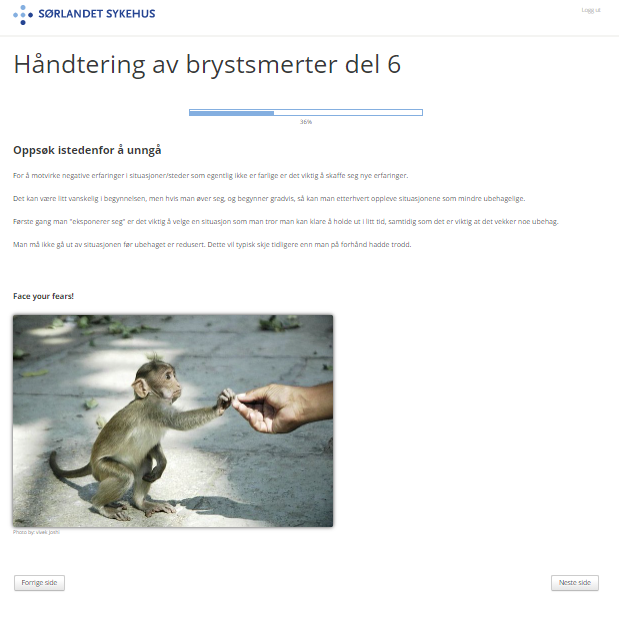


**Snapshot 8:** Planning of homework assignments between session 3 and 4


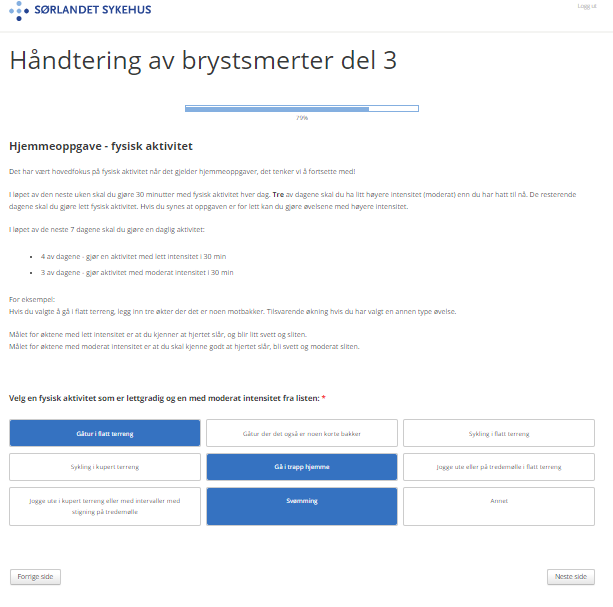


**Session 9:** Reporting homework assignments electronically
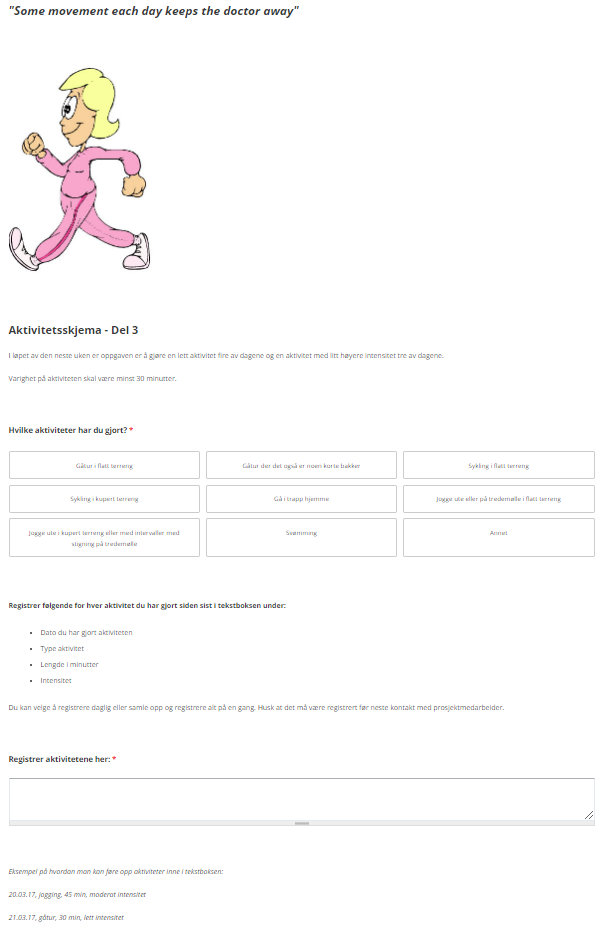

Supplement: Multimedia Appendix 1 [file jmir_v24i1e33631_app1.docx]
